# 5 years of *The Lancet Regional Health – Americas*

**DOI:** 10.1016/j.lana.2026.101639

**Published:** 2026-09-10

**Authors:** Taissa Vila, Gustavo Monnerat, Orison Woolcott, Maria Dario

5 years ago, on Sept 1, 2021, the first issue of *The Lancet Regional Health – Americas* was published. With 12 research Articles, six Comments, and one Viewpoint, we launched a platform to give voice to the Americas. 5 years later, we reflect on what we have achieved, what we have learned from the region, and where we want to go next.

Launching a journal in the middle of the COVID-19 pandemic was a unique challenge, but also an invaluable opportunity. Since our first issue, we sought to contribute to building knowledge about COVID-19 and supported rigorous science produced in the region to guide evidence-based decisions: early research provided real-world evidence on CoronaVac effectiveness during gamma-variant transmission, while another study estimated that at least 303,129 lives were saved during the first year of COVID-19 vaccination in Brazil, supporting continued vaccine deployment. We also used our platform to counter misinformation as the region—and the whole world—navigated uncertainty.

The pandemic exposed deep-rooted structural inequities and broken health-care systems across the Americas. It showed how differences in access to health care, economic security, scientific capacity, and public-health infrastructure can determine who is protected and who is left behind. These inequities are shaped by intersecting social, commercial, and political determinants of health, the same determinants that define who is most exposed to and least protected from the ongoing climate crisis. Since 2023, reports by the *Lancet Countdown* document climate-health evidence from Latin America to inform public decision making, especially preparedness, mitigation, and adaptation plans. The Americas is increasingly exposed to extreme heat, floods, fires, droughts, infectious diseases, food insecurity, and population displacement. In all of those, systematically marginalised populations are often disproportionately affected. Our Commission on primary health-care resilience in Latin America and the Caribbean highlighted the need to build health systems that can maintain essential services before, during, and after shocks.

In our inaugural Editorial, we pledged to give a voice to research scientists, clinicians, policy makers, and other stakeholders by creating an open forum for constructive discussions to advance research and health care in the region. Importantly, we aimed to build a platform to increase the visibility of regionally important questions being investigated by locally based researchers. Over the past 5 years, Latin American and Caribbean researchers have appeared on 56.9% of our published research Articles and account for 38.7% of corresponding authorships. Women are 49.9% of research first authors and 53.9% of review first authors. Our output has grown alongside interest in the journal: from 19 pieces on our first issue to around 25 per issue today, supported by an editorial team that has expanded with two new editors.

Over these 5 years the journal has covered a wide range of regionally driven topics such as cardiovascular health (including a series on Stroke prevention) infectious diseases, diabetes, maternal and neonatal health, migration and displacement, the opioid crisis, health financing, and primary care. Studies on front-of-pack nutrition labelling and implementation of WHO hypertension guidance show how applied regional evidence can support policy decisions. We have also supported Indigenous authority and self-determination over the research, knowledge, and data systems that shape Indigenous health. Celidwen and colleagues’ paper on traditional Indigenous medicine called for Indigenous knowledge, rights, and sovereignty to be central to Western psychedelic research and practice, and Govorchin et al. called for Indigenous data sovereignty in Canada.

The scientific landscape has changed considerably since 2021. Political attacks on science, vaccines, public-health institutions, and diversity, equity, and inclusion have created a more difficult environment for researchers and for the production and use of evidence. In our most cited paper to date, Turner et al. showed persistent shortcomings in race and ethnicity reporting and representation in US clinical trials, an inequity with direct implications for whose health is counted in research and policy. A comparable investigation proposed today could face greater scrutiny in a US federal research environment that has targeted equity-related programmes and narrowed official terminology in research. The consequences of such developments extend beyond the Americas, threaten research partnerships, weaken institutions, discourage researchers working on inequity and marginalised populations, and create opportunities for misinformation to shape the public debate. In this context, the journal's role cannot be limited to documenting inequities but must continue to defend independent, inclusive science, and create space for evidence that can support more equitable health policies and systems.

As we look to the next 5 years, we want the journal to move from giving the region a voice to pressing for regional evidence to be heard, trusted, and acted upon. We will continue to support locally relevant and led research, strengthening work on the social, commercial, and political determinants of health and the policies and systems that shape health outcomes. We will seek to amplify perspectives that remain absent from scientific debates and continue to advocate for universal, high-quality, and accessible health care with the goal of improving outcomes and reducing health inequity in the Americas.

This milestone belongs to the community that has helped shape the journal since its first issue: our authors, peer reviewers, international advisory board, readers, and listeners. We thank them for the rigour, generosity, and engagement they continue to bring through each contribution.

